# Situating Language in the Real-World: Authors’ Reply to Commentaries

**DOI:** 10.5334/joc.181

**Published:** 2021-08-23

**Authors:** Margherita Murgiano, Yasamin Motamedi, Gabriella Vigliocco

**Affiliations:** 1 Experimental Psychology, University College London, GB; 2 Centre for Language Evolution, University of Edinburgh, GB

**Keywords:** iconicity, multimodal communication, language acquisition, language processing

## 1. Broadening our view of language

In promoting an ecologically valid approach to the study of language and, more specifically, non-arbitrariness, our paper distinguished between two perspectives from which language can be studied: the ‘language as a system’ and the ‘language as situated’. These two perspectives do not stand as alternatives; rather, in our view, the *language as situated* encompasses the *language as a system* perspective. It is a more comprehensive approach that includes all the linguistic components traditionally studied but also the multimodal, often non-arbitrary, cues that are overwhelmingly present *in the moment* in language use. These cues characterize the way in which language is concretely learnt and understood in the real-world. Thus, it is not surprising that, as noted by **Perlman and Woodin**, iconicity, which is a key feature of the *language as situated* view, is also present and maintained in the *language as a system* view. Or, as argued by **Ozyurek**, that systematicity and language specificity, key features of the *language as a system* perspective, are also found in gestures which are part of language only under a situated perspective.

The distinction between ‘system’ and ‘situated’ does not coincide with the distinction between rule-governed, conventional elements of language, and universally accessible components of communication devoid of any conventionality as implied in **Ozyurek’s** commentary. Situated language refers to the real-world uses of language rather than universality. We argue in favor of the importance of reintegrating the observation of language use in any theoretical and empirical approach to language study. In such an approach, language cannot be divorced from the *physical* and *communicative context* or from cultural, individual and linguistic differences and iconicity cannot be thought of as an universal and direct mapping between a sign and an object. Multimodal iconic cues exploit a motivated correspondence between form and meaning through a selection of those semantic aspects that are salient in a certain physical, cultural and linguistic context. We argue that this contextually motivated mapping between form and meaning facilitates the access to the latter and consequently helps language acquisition and processing.

## 2. Iconicity in the *system* and iconicity in the *situated* view

We fully agree with **Perlman and Woodin** that iconicity is present in those systemic and categorical parts of language in far larger extent than it has often been acknowledged in linguistic research (Hockett, 1960; Newmeyer, 1992) and that it is actively maintained (see also Vinson et al., 2021). However, the amount of iconicity is still going to be vastly underestimated unless a *language as situated* view is taken. Multimodal non-arbitrary cues such as gestures and prosodic modulations not only provide additional sources of iconicity, but can further modify and extend iconicity in the system. For example, research on ideophones has highlighted that the totality of their iconic expression cannot be attributed to sound symbolism in the lexeme/morpheme alone, but in how it is used in combination with other cues such as prosody and iconic gesture (Dingemanse, 2012; Nuckolls, 1996), and that playful language use regularly creates or enhances iconic expression in context (Dingemanse and Thompson, 2020; Jakobson and Waugh, 1979; Kaneko and Sutton-Spence, 2012).

Why would iconicity be maintained in the systemic aspects of language? We suggest that iconicity is maintained because it might be beneficial in ways that are not confined to early language emergence or first language acquisition, but that represent everyday communication, for example in grounding communication, either for language learners without existing conventions or mature language users navigating a new communicative context, with iconicity being, as we explained above, not a fixed property but one that can be modulated and made salient in context.

Also, some degree of iconicity in the system (allowing for a more transparent mapping between communicative form and meaning) may simply be necessary for communicative success in non-situated contexts like when written language is used. Using methods such as lexical decision or semantic categorization on written words (as discussed by **Sidhu and Pexman**) taps into this type of baseline iconicity. Sidhu and Pexman also discuss the possibility that the iconicity addressed in studies of written language also tap into potential for iconicity. We disagree with this because we can have words that have very low iconicity ratings becoming iconic *in situ* by virtue of gestures or iconic prosody or sound effects, while the iconicity of words with high iconicity ratings is not necessarily increased in face-to-face communicative contexts.

We cannot agree more with **Emmorey** who says that historically, the perspective that draws a strict line between *system* and *situated* comes from looking at spoken languages (see also Vigliocco et al., 2014). If the study of language started from the investigation of signed languages, not only would we not draw such a line, in addition we would see the *system* as part of the *situated*. She offers the perfect example of why this would be the case: the production of lexical signs in close connection with drawings or written words, environmental elements that reinforce and further explain the meaning of those signs through physical connection. We argue that spoken languages can also have a similar level of connection with external, environmental factors: we can observe an example of this with onomatopoeia, that can be articulated along with other environmental noises or sounds, or with iconic prosody with which we can for example express the length of an object while following its shape with our hand.

## 3. *Language as situated* and Embodiment

What is the relationship between the broader perspective provided by the situated view of language and embodiment? This question was not discussed in the target article, but clearly is an important issue to address in the future. Our hypothesis is that the extent to which comprehenders need to simulate (i.e., develop imagistic representations based on their knowledge) what is being said depends on the level of situatedness of the communicative situation. The physical context necessarily matters in determining whether the comprehender needs to simulate: if the comprehender is watching a cooking show in which the speaker (the chef) is describing what they are doing while they are doing it, there will be less need to develop internal simulations. However, if the comprehender reads a novel, there will not be any support from the physical context and therefore greater need to develop internal simulations (Zwaan, 2014). Taking the *language as situated* perspective, the need (and presumably amount) of simulation that a comprehender will develop will also depend on the presence of non-arbitrary cues provided by the speaker, further reducing the need for internal simulations. Thus, words and phrases can become meaningful by virtue of eliciting the retrieval of sensorimotor and affective information from memory, as argued by **Long, Chia and Kaschak**. However, this may be the case only when neither the *physical*, nor, crucially, the *communicative* context provide access to sensorimotor and affective information in a more direct manner via indexical and iconic cues.
